# Targeting Transcription Through Inhibition of TBP

**DOI:** 10.18632/oncotarget.226

**Published:** 2011-03-01

**Authors:** Thaddeus T. Schug

**Affiliations:** National Institute of Environmental Health Sciences, Division of Extramural Research and Training, Cellular, Organ and Systems Pathobiology Branch, Research Triangle Park, NC 27709, USA

**Figure 1:**
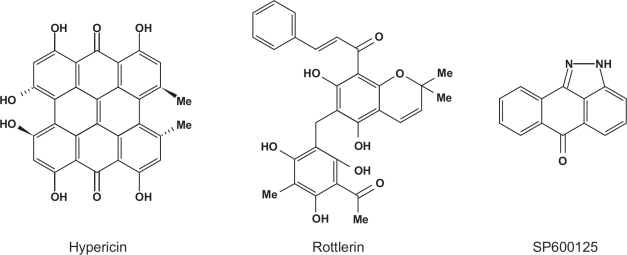
Structural representations of the kinase inhibitors Hyericin, Rottlerin, and SP600125 Hyerpericin is an antibiotic and one of the principle ingrediants in Saint John's wort. Rottlerin is extracted from the Kamala plant *Mallotus philippinensis*, and is a widely-used selective inhibitor of protein kinase C. SP600125 is inhibitor of c-Jun N-terminal kinase (JNK).

Transcriptional regulation by RNA polymerase II (RNA Pol II) is a highly regulated process involving the action of multiple transcription factors that collectively regulate synthesis of messenger RNA (mRNA). These complexes sit atop core promoter regions of DNA composed of sequence motifs that mark the starting point of transcription [1]. Studies have shown that the transcriptional machinery is capable of sensing changes in cellular stress levels and directing cell fate by modulating production of genes involved in cell cycle regulation [2]. Very few molecules are capable of directly targeting transcription, making this an area of intense therapeutic interest. In this issue of *Oncotarget*, Morachis et al. describe identification of three distinct drugs that inhibit mRNA synthesis [3]. Surprisingly, these drugs, Hypericin, Rottlerin, and SP600125 (Figure 1), are kinase inhibitors that impair transcription initiation by targeting components of the Pol II pre-initiation complex. A common target of these compounds is the inhibition of the TATA binding protein which helps position Pol II along the transcription start site of p53-responsive promoters. Development of this new methodology, together with identification of these compounds as transcriptional inhibitors, will pave the way toward providing mechanistic insight into cause and treatment of disease.

**Figure 2:**
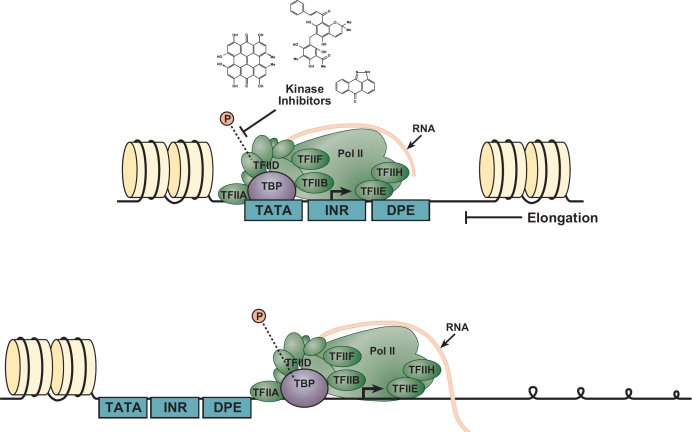
The multi-subunit general transcription apparatus PIC assembly begins with subunits recognizing the core promoter elements, followed by coordinated accretion of the complex machinery. TBP binding to the TATA box is an intrinsically slow step, yielding a long-lived protein–DNA complex. TBP phosphorylation is necessary to enable the complex to move from the initiation (top image) to the elongation phase of transcription (bottom image). Kinase inhibiting compounds such as Hypericin, Rottlerin, and SP600125, prevent phosphorylation of TBP thus preventing the Poll II complex from progressing to the elongation phase.

The p53 tumor suppressor protein promotes longevity by reducing somatic mutations or the survival and proliferation of mutant cells [4]. Almost all human cancers contain impairments in the p53 signaling pathway. Currently, intense focus centers on understanding how p53 chooses which of its multiple target genes to activate or repress in response to a given stress. A potential source enabling the diverse functions of p53 lies within the core promoter regions of its target genes [5]. The core promoter, where transcription is initiated, contains conserved motifs such as TATA box, initiator (INR), TFIIB recognition element (BRE), down-stream promoter element (DPE) and down-stream core element (DCE) (Figure 2) [6]. Morachis et al. analyzed the impact of multiple kinase inhibitors on RNA transcriptional activity along three p53 target gene core regulatory elements using transcription and electrophoretic mobility shift (EMSA) assays. And, while other drugs such as Flavopiridol were shown to abolish transcription during the elongation phase [7], this study reports that Hypericin, Rottlerin, and SP600125 inhibit modification of the TATA Binding Protein (TBP) during the initial phase of transcription. Importantly, the study further determines that the three compounds inhibit TBP phosphorylation on both TATA box-containing and TATA-less promoters. A previous study demonstrated that yeast TBP could be phosphorylated by the protein kinase CK2, thus reducing its affinity to the TATA element [8], thus offering a potential mechanism of action. In summary, these findings add to the limited toolbox available to scientists studying transcriptional regulation and may aid in development of new therapeutic candidates to treat disease.

The same group has recently demonstrated that p53 target promoters are structurally diverse and display pronounced differences in Poll II occupancy [9]. Their studies reveal that, far from being a latent tumor suppressor, p53 functions in a temporal manner to regulate promoter activity both before and after cellular stress. This is achieved by the ability of p53 to establish markedly different affinities of Poll II on its diverse target promoters and recruit transcriptional initiation components in a stress-specific manner. It will be important to determine how p53 directs the recruitment of Poll II and specific cofactors to its diverse target promoters before and after stress to generate the appropriate transcriptional response and how this process fails in human cancers.
